# Effect of sodium diffusion on the properties of CIGS solar absorbers prepared using elemental Se in a two-step process

**DOI:** 10.1038/s41598-019-39283-2

**Published:** 2019-02-25

**Authors:** Weimin Li, Xia Yan, Armin G. Aberle, Selvaraj Venkataraj

**Affiliations:** 1 Solar Energy Research Institute of Singapore, National University of Singapore, Singapore, 117574 Singapore; 2 Department of Electrical and Computer Engineering, National University of Singapore, Singapore, 117576 Singapore; 3 Center for Information Photonics and Energy Materials, Shenzhen Institutes of Advanced Technology, Chinese Academy of Sciences, Shenzhen, 518055 China

## Abstract

The influence of Na diffusion from various glass substrates during a high-temperature slenization process on the microstructure and morphology of two-step formed CIGS absorber layers is investigated. In order to minimise the CIGS absorber formation time, elemental Se vapour is used to prepare the CIGS absorber. The grain sizes of the CIGS films are found to increase with increasing sodium in the glass substrates (extra clear glass, soda-lime glass, borosilicate glass). TiN and SiN thin films are used as diffusion barrier layers inserted between the glass substrate and the Mo rear conatct to tune the Na diffusion from the soda-lime glass. The interdiffusion between the In-rich CuInSe_2_ surface layer and the Ga-rich CuGaSe_2_ layer is promoted by the barrier layer, leading to larger CIGS grains. Efforts are also taken to understand the differences in Na diffusion (from the glass substrates) and their effects on the MoSe_2_ intermediate layer formation during the high-temperature CIGS absorber formation processes. We find that excess amounts of Na and Se are essential for the MoSe_2_ growth. The excessive Na in the form of Na_2_Se_x_ at the CIGS/Mo interface works as a Se source and catalyses the MoSe_2_ formation. The Se flow in the two-step CIGS formation process must be sufficiently high to obtain high-efficiency CIGS solar cells.

## Introduction

The copper indium gallium di-selenide (CIGS) solar cell is one of the most efficient solar cells for standard 1-sun application, with efficiencies of up to 22.9% at the lab scale using a very thin absorber layer (<3 μm)^1^. Molybdenum (Mo) thin films deposited by magnetron sputtering is the most widely used rear contact for CIGS photovoltaic, as it can meet most of the back contact requirements and ensures high-efficiency CIGS solar cells^2–6^. Mo films deposited by magnetron sputtering show low electrical resistivity (sheet resistance <0.5 Ω/sq), high thermal stability, and good corrosion resistance during the CIGS absorber formation process which normally takes place at a high temperature of 500–600 °C^7–11^. In addition, Mo film also offers a low resistive ohmic contact between the Mo back contact and the CIGS absorber by forming a thin MoSe_2_ layer at the Mo/CIGS interface during the high temperature absorber formation step^12–14^.

During the two-step CIGS formation process, Na diffusion from the substrate plays an important role in forming a uniform CIGS film: the Na content can postpone the inter-diffusion of In and Ga atoms to form a uniform CIGS layer^15–19^. However, the Na diffusion is related to the properties of the rear contact and the selenization process. The rear contact properties have a strong influence on the formation of the intermediate MoSe_2_ layer and the properties of the formed CIGS absorber layer^12,20,21^. The MoSe_2_ layer plays a significant role in ensuring a low contact resistance and good adhesion of the CIGS absorber to the rear contact. The CIGS layer tends to delaminate from the Mo coated substrate after CdS buffer layer deposition if its adhesion to the substrate is not promoted by the MoSe_2_ layer, which is normally formed at the Mo and CIGS interface during high temperature CIGS formation process. The existence of a thin MoSe_2_ layer can decrease the apparent Schottky barrier height and thereby provide a better ohmic contact to the CIGS absorber. Simultaneously, MoSe_2_ is able to enhance the mechanical peel strength of CIGS to Mo back contact. However, an excessive thickness of MoSe_2_ layer formed at the Mo and CIGS interface is reported to deteriorate the performance of the CIGS solar cells due to the high resistivity of MoSe_2_ (10^1^–10^4^ Ohm-cm)^14^. Formation of MoSe_2_ depends mainly on the selenisation process and alkali Na diffusion from the soda-lime glass (SLG) substrate. Moreover, the preferred orientation of the MoSe_2_ crystal grains is important for the adhesion of the CIGS layer to the rear contact. Hence, controlling the MoSe_2_ layer formation and thickness are important for preparing high-efficiency CIGS solar cells.

In this paper, the selenisation of the metallic Cu-In-Ga precursor was carried out in nitrogen atmosphere at ambient pressure using elemental Se vapour by an inline rapid thermal processing furnace (Smit Thermal Solutions, Netherlands). There are many advantages in using elemental Se vapour instead of toxic H_2_Se gas as the Se source, such as lower cost, easier handling and shorter absorber process time of <15 minutes (which is approximately 3 times faster than the conventional CIGS formation using H_2_Se gas). In this work, efforts are taken to understand the difference in Na diffusion (from the SLG substrate) and their effects on MoSe_2_ intermediate layer formation during the high-temperature CIGS absorber formation process. Bilayer Mo rear contacts are deposited on glass substrates containing different sodium content.

## Experimental Details

**Figure 1:**
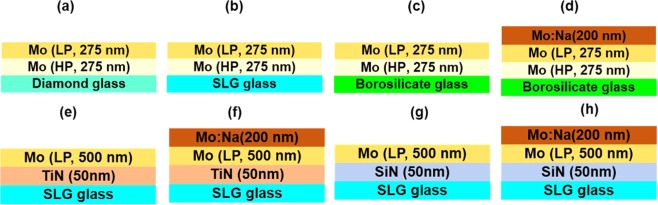
Different back contact designs used for CIGS solar cell fabrication: (**a)** Dia/Mo, (**b**) SLG/Mo, (**c**) Boro/Mo, (**d**) Boro/MoNa, (**e**) SLG/TiN/Mo, (**f**) SLG/TiN/MoNa, (**g**) SLG/SiN/Mo, and (**h**) SLG/SiN/MoNa.

The different glass substrates and rear contact designs utilized in this study for CIGS solar cell fabrication are shown in Fig. 1. The optimized deposition conditions of the individual layers used to form various back contact stack designs are discribed in our previous publication^18^. Bilayer Mo films comprising of a high pressure (HP, 6.0 × 10^−3^ mbar) deposited bottom Mo layer and a low pressure (LP, 1.5 × 10^−3^ mbar) deposited top Mo layer are deposited on three different glass substrates (size: 30 cm × 40 cm) with different Na concentrations: extra clear glass (solar grade opti-white diamond glass, Dia, high Na concentration), soda-lime glass (SLG, intermediate Na concentration), borosilicate glass (Boro, negligible Na concentration), see Fig. 1(a–c)^22–24^. On Na-free borosilicate glass substrate, for comparative purposes, an additional 200 nm thick Mo:Na (3 wt%) film is deposited on top of the bilayer Mo stack, which serves as an extrinsic Na source (see Fig. 1(d)). In addition, TiN and SiN thin films are utilised as sodium diffusion barrier and adhesion enhancement layer for the LP-deposited 500-nm Mo film onto SLG substrates (see Fig. 1(e,g))^19^. Finally, a 200 nm thick Mo:Na layer (see Fig. 1.1(f,h)), used as extrinsic Na source, is deposited onto these rear contacts with a thin TiN or SiN sodium diffusion barrier layer.

The Mo films using different back contact designs were cut into 10 cm × 10 cm substrates for CuGa/In precursor deposition. A four-layer CuGa/In/CuGa/In stack design was used to prepare the Cu-In-Ga precursors by direct current (DC) magnetron sputtering. The selenisation of the precursors was performed in nitrogen atmosphere at ambient pressure condition. The heater temperatures of the RTP (rapid thermal process) were 580 °C and the duration of the high temperature process was about 12 minutes. Thermally evaporated (420 °C) Se vapour was used as the Se source to form CIGS absorber layers. The average GGI ([Ga]/([Ga] + [In])) and CGI ([Cu]/([Ga] + [In])) ratios of the CIGS absorber measured by X-ray Fluorescence (XRF) were about 0.30 and0.93, respectively. Solar cells with these CGI and GGI ratios are reported to achieve high efficiencies of above 20%^25–27^.

## Results

**Figure 2:**
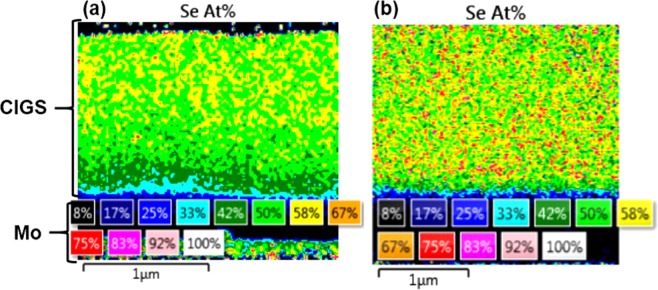
Cross-sectional SEM-EDX Se elemental mapping of CIGS absorbers formed on SLG/Mo substrates with different Se contents: (**a**) Se-poor (50 at.% measured by XRF) and (**b**) Se-rich (57 at.% measured by XRF). The different colours at the bottom denote the corresponding atomic percent values.

In this study, CIGS absorbers with different Se contents (50 and 57 at.%) were fabricated via changing the Se crucible temperature in order to investigate the effect of the Se content on the formation of the MoSe_2_ layer. Figure 2 shows Scanning electron microscopy with energy dispersive X-ray spectroscopy (SEM-EDX) images of Se elemental mapping (at.%) across the absorber layer thickness. It can clearly be seen that the Se content in Fig. 2(a) decreases gradually from the top to the bottom of the absorber. At the rear surface of the absorber, i.e. at the Mo/CIGS interface, an approximately 200 nm thick CIGS layer was found to contain about 33% to 42% of Se, indicating a Se-poor absorber. However, for the sample shown in Fig. 2(b), the Se is distributed uniformly at above 50 at.%, showing a Se-rich absorber. The effects of the different rear contacts on the properties of the Se-poor and Se-rich CIGS absorber regions are investigated and discussed in the following sections.

### Se-poor CIGS absorber

#### Surface morphology

**Figure 3:**
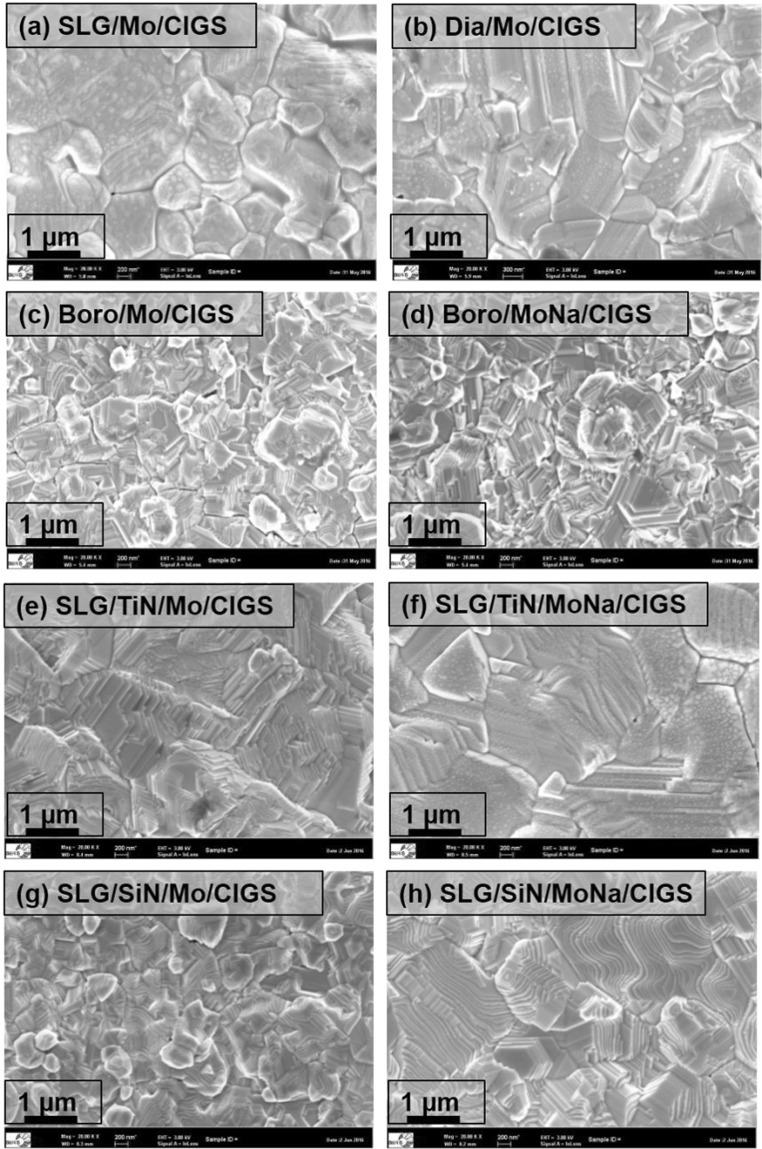
Surface SEM images of CIGS absorbers formed on (**a**) Dia/Mo, (**b**) SLG/Mo, (**c**) Boro/Mo, (**d**) Boro/MoNa, (**e**) SLG/TiN/Mo, (**f**) SLG/TiN/MoNa, (**g**) SLG/SiN/Mo, (**h**) SLG/SiN/MoNa.

The surface morphology of the CIGS films formed on different rear contact structures was studied by SEM, see Fig. 3. The surface grain boundaries of Boro/Mo/CIGS and SLG/SiN/Mo/CIGS samples are clearly observed in Fig. 3(c,g), while white Na_2_Se precipitates are found on the surface of SLG/TiN/Mo/CIGS samples and samples having a Mo:Na layer at the rear contact as well.

The grain size of the CIGS films formed on bilayer Mo-coated diamond glass and soda-lime glass was much larger than that of the CIGS film formed on borosilicate glass, which was attributed to Na diffusion from the glass substrate through the bilayer Mo rear contact into the absorber. Thus, Na can diffuse through the voids between the columnar structures of bilayer Mo films. The addition of a 200 nm thick Mo:Na layer on top of the bilayer Mo rear contact slightly increased the grain size of the CIGS film. For the modified rear contacts deposited on SLG substrates, the grain size of the CIGS films formed on SLG/TiN/Mo rear contacts was much larger than that of CIGS films formed on SLG/SiN/Mo rear contacts (see Fig. 3(g)). In addition, the grain sizes of the Mo films deposited on SLG/SiN/Mo and on Boro/Mo substrates were quite similar (in the 0.5–1 μm range). These two findings indicate that a SiN barrier layer is more efficient in terms of blocking the Na diffusion from the soda-lime glass substrate than a TiN barrier layer. Using a Mo:Na film as extrinsic Na source, the grain size of the CIGS films formed on SLG substrates with a TiN or SiN barrier layer were in the same range (about 1–3 μm) as those without a barrier layer in the rear contacts.

**Figure 4:**
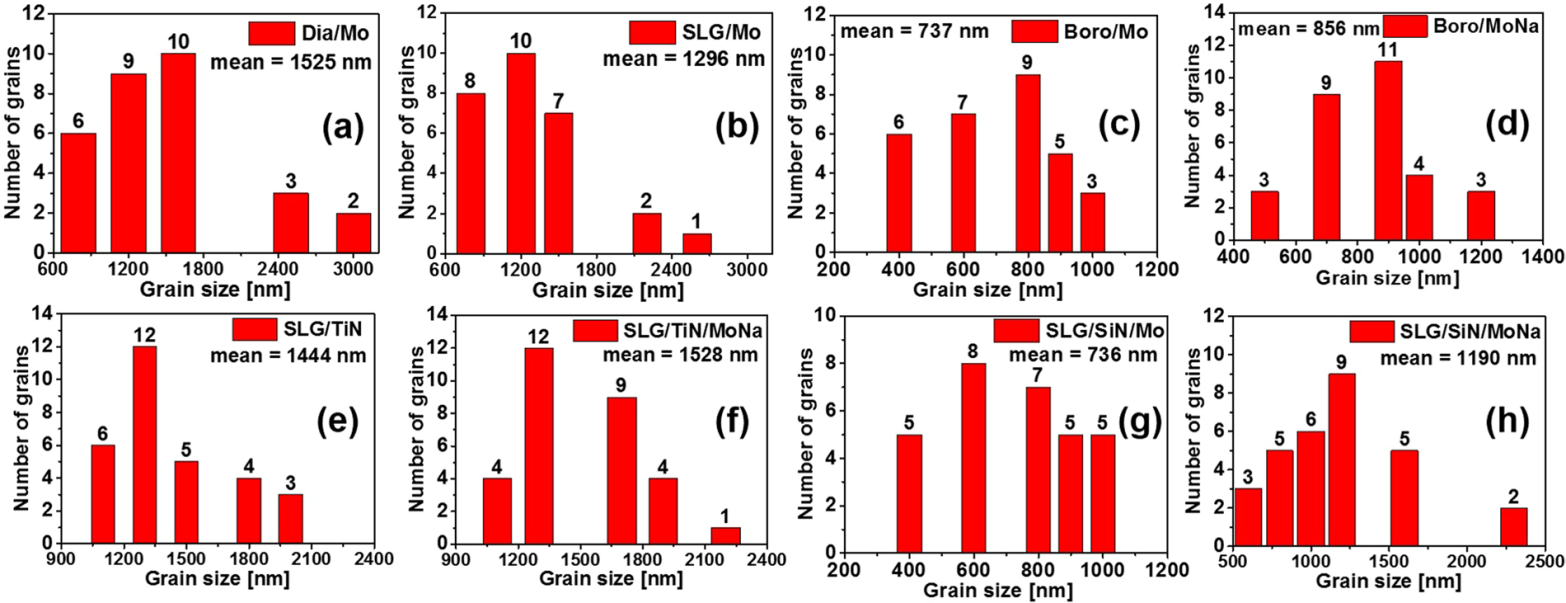
Histogram plot of the CIGS absorber grain size formed on (**a**) Dia/Mo, (**b**) SLG/Mo, (**c**) Boro/Mo, (**d**) Boro/MoNa, (**e**) SLG/TiN/Mo, (**f**) SLG/TiN/MoNa, (**g**) SLG/SiN/Mo, (**h**) SLG/SiN/MoNa.

The grain sizes of the CIGS absorber were measured via surface SEM images. Histogram plots of the CIGS absorber grain size formed on different glass substrates and rear contacts are depicted in Fig. 4. The calculated average grain sizes are also shown in Fig. 4. The SEM studies clearly reveal that the Na incorporation either from the glass substrate or from the Mo:Na layer can enhance the CIGS grain size. It should also be noted that the CIGS film deposited on the SLG/TiN/Mo substrate showed the best lateral uniformity.

#### Chemical composition

The XRF measurement results for the CIGS absorber with 50 at.% Se formed on different rear contacts are shown in Table 1. The SeC and SeGI are [Se]/[Cu] and [Se]/([Ga] + [In]) ratios. The average CGI and GGI ratios of these CIGS absorbers are about 0.93 and 0.30. The calculated Se/M ([Se]/([Cu] + [In] + [Ga]) ratio was slightly higher than 2.0. It is reported in the literature that the excess Se in CIGS solar cells exists in the form of a MoSe_2_ layer^20^. The thickness of the MoSe_2_ (${t}_{MoS{e}_{2}}$) layer can be estimated from the following equation^20^:

1 $${t}_{MoS{e}_{2}}={t}_{CIGS}\frac{({c}_{Se}^{exp}-{C}_{Se}^{nom})}{{C}_{Se}^{MoS{e}_{2}}}\frac{{n}_{CIGS}}{{n}_{MoS{e}_{2}}}\,$$

where ${C}_{Se}^{exp}$ is the atomic percentage of Se in the absorber as measured by XRF, ${C}_{Se}^{nom}$ is the nominal atomic percentage of Se in the absorber,$\,{C}_{Se}^{MoS{e}_{2}}$ is the atomic percentage of Se in the MoSe_2_ (i.e. 0.67), ${n}_{CIGS}$ is the atomic density of CIGS (i.e. 4.23 × 10^22^ cm^−3^), and ${n}_{MoS{e}_{2}}$ is the atomic density of MoSe_2_ (i.e. 5.15 × 10^22^ cm^−3^). The nominal atomic percentage of Se in the absorber ${C}_{Se}^{nom}$ is calculated by:

2 $${C}_{Se}^{nom}=\frac{1+3/x}{3+5/x}$$

where *x* is the GGI ratio ([Ga]/([Ga] + [In]) in the absorber.

**Table 1:** Summary of XRF measurement results for Se-poor CIGS absorbers formed on different glass substrates and rear contacts.

Sample number	CGI	GGI	SeC	SeGI	Se/M	Mo	MoSe_2_	CIGS
						[nm]	[nm]	[nm]
Boro/Mo	0.92	0.31	2.41	2.22	1.15	574	54	1375
Boro/MoNa	0.94	0.31	2.46	2.31	1.19	742	54	1380
Dia/Mo	0.93	0.31	2.59	2.41	1.25	562	55	1412
SLG/Mo	0.93	0.31	2.47	2.30	1.19	550	54	1380
SLG/TiN/Mo	0.91	0.30	2.48	2.25	1.18	504	54	1375
SLG/TiN/MoNa	0.89	0.30	2.47	2.21	1.17	749	54	1363
SLG/SiN/Mo	0.93	0.31	2.40	2.23	1.16	482	54	1392
SLG/SiN/MoNa	0.93	0.30	2.47	2.28	1.19	698	54	1336

The MoSe_2_ layer thickness is estimated using Eq. 1 and assumed to be constant for all samples.

The thickness of the MoSe_2_ layer estimated using Eq. 1 is listed in Table 1. The estimated thicknesses of the MoSe_2_ layers of all the Se-poor samples are quite similar (∼54 nm). Therefore, it is not possible to derive a correlation between the MoSe_2_ layer thickness and the Na content, as the thickness of the MoSe_2_ intermediate layer is the same regardless of the Na content in the glass substrates. Also, it is not possible to visibly observe the MoSe_2_ layer at the Mo/CIGS interface in the cross-sectional SEM measurements^18^, which indicates that the estimated MoSe_2_ layer thickness (54 nm) from Eq. 1 is reliable. Thus, the Na concentration is not the only factor that affects the formation of the MoSe_2_ layer during the high-temperature selenisation process.

However, it should be noted that the Se/M ratio in the CIGS absorber is higher for SLG/Mo and extra clear (diamond glass)/Mo rear contacts as compared to those with Boro/Mo and SLG/TiN/Mo (or SLG/SiN/Mo) rear contacts. For example, the Se/M ratio of the absorbers formed on Dia/Mo substrates was the highest (1.25) of the three investigated glass substrate types. Thus, Na can assist the incorporation of Se into the absorber by the formation of Na_2_Se_x_. It should also be noted that the thickness of the CIGS layer increased with increasing Na content in the glass substrate, which confirms that a certain percentage of Na_2_Se is always present at the CIGS grain boundaries, and also at the Mo/CIGS interface, which was confirmed by the SIMS measurements^18^.

#### Microstructure

**Figure 5:**
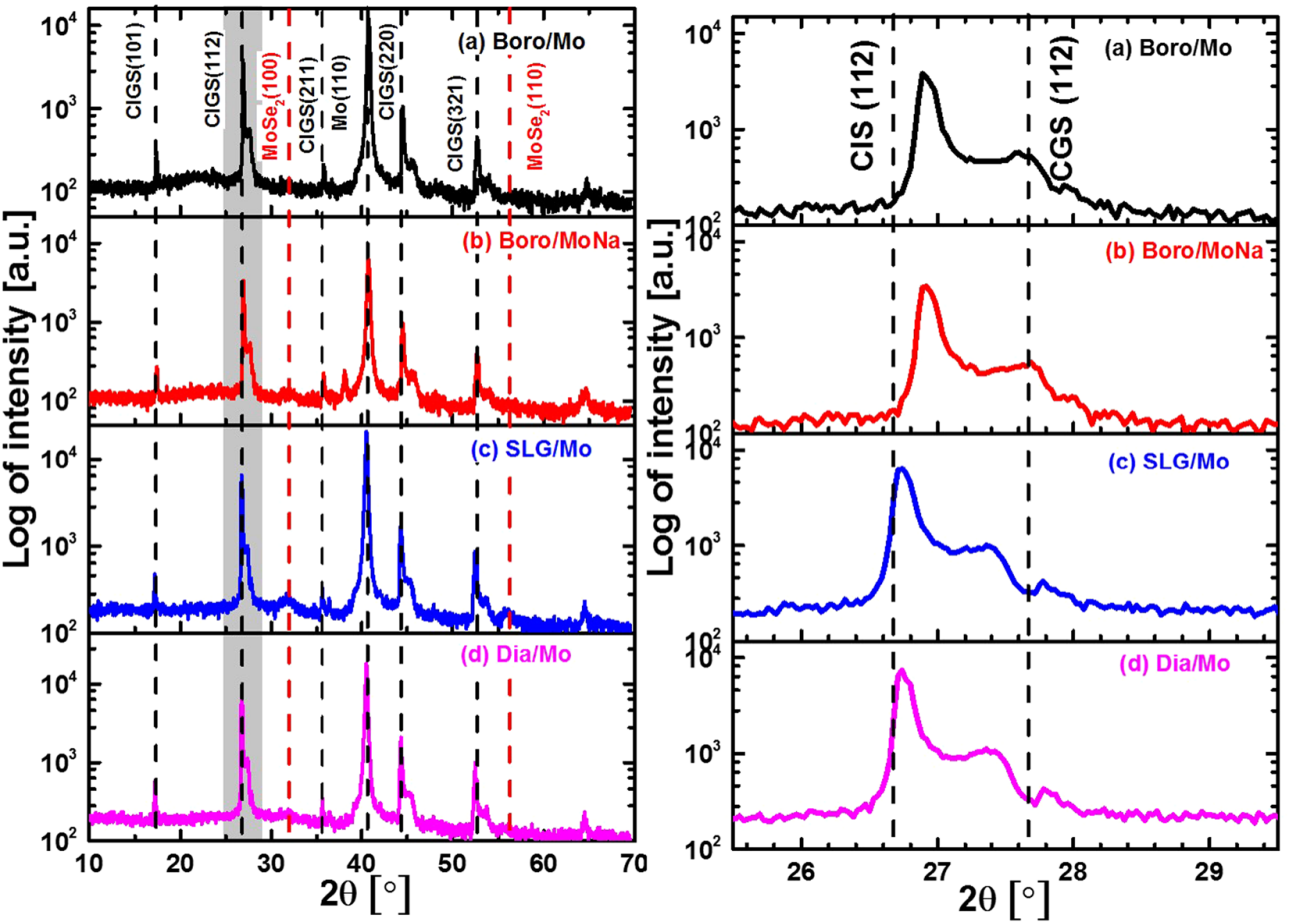
XRD patterns and the CIGS (112) peaks of CIGS absorbers deposited on different glass substrates: (**a**) Diamond/Mo, (**b**) SLG/Mo, (**c**) Boro/Mo, and (**d**) Boro/MoNa. Attributions of each XRD peak are indicated in the plot as well.

**Figure 6:**
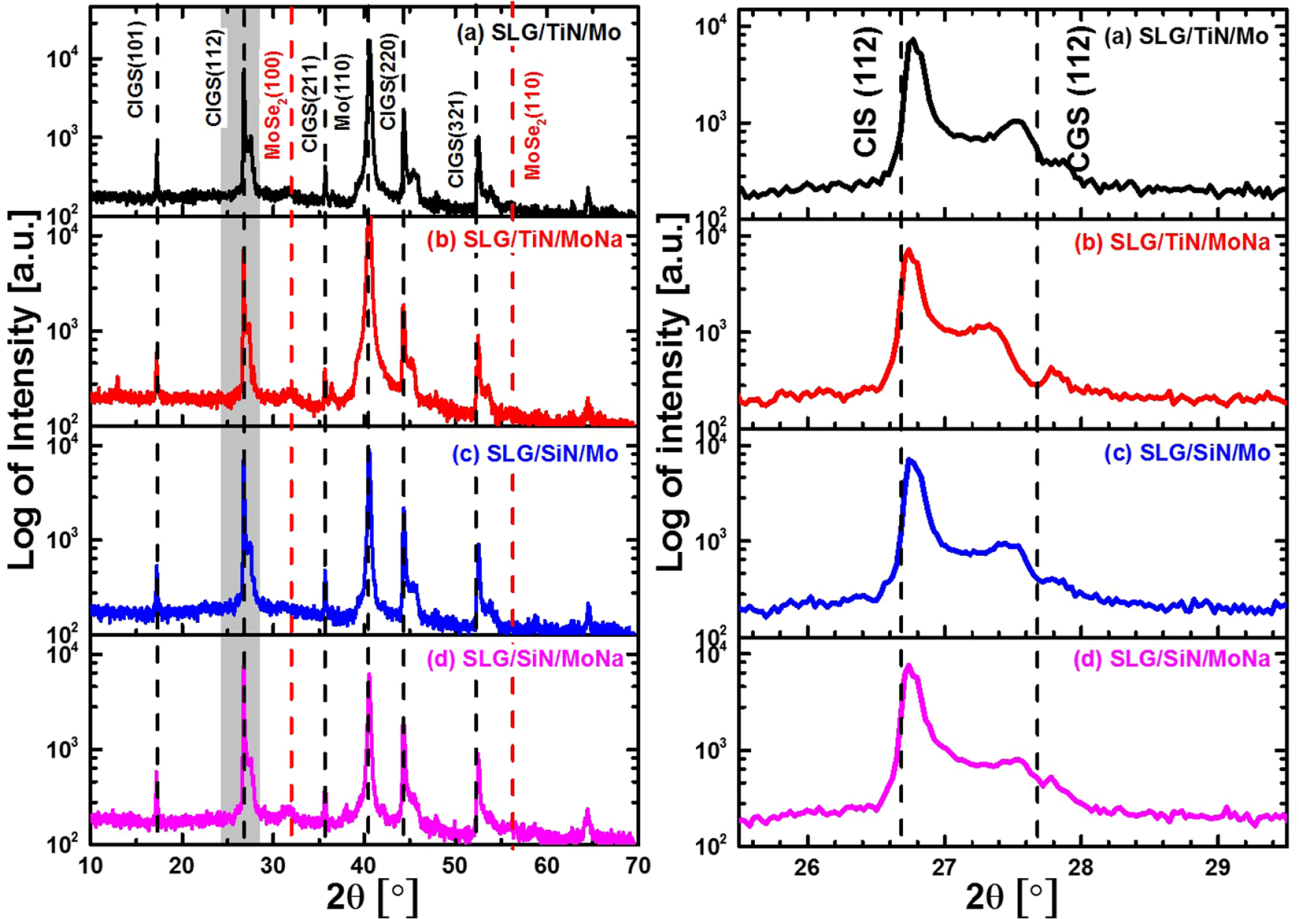
XRD patterns and the CIGS (112) peaks of CIGS absorbers deposited on different modified rear contacts: (**a**) SLG/TiN/Mo, (**b**) SLG/TiN/MoNa, (**c**) SLG/SiN/Mo, and (**d**) SLG/SiN/MoNa.

The XRD patterns and the CIGS (112) peak profiles of the Se-poor CIGS absorbers deposited on glass substrates containing different sodium content and rear contacts are shown in Figs 5 and 6. The peak position, peak intensity and other important parameters derived from XRD measurements are summarised in Table 2. As shown in Figs 5 and 6, all CIGS films show a polycrystalline structure, indicating the formation of stoichiometric CIGS. Moreover, there is no diffraction peak at 2θ = 26.2° indicating that Cu_2−x_Se secondary phase was not formed on the surface of the CIGS absorber. The high-intensity peak of the Mo (110) orientation was found at 2θ = 40.5°. The intensity ratio I(220/204)/I(112) shown in Table 2 was calculated to evaluate the crystal growth of the CIGS films. There was little variation in the I(220/204)/I(112) ratio between the samples prepared on different glass substrates and rear contacts. A slight reduction in the (220/204) peak intensity was observed in samples deposited on SLG/TiN/MoNa and SLG/SiN/MoNa substrates compared to those without Mo:Na layer, which was attributed to the extrinsic Na incorporation from the Mo:Na layer^28^.

**Table 2:** Summary of the XRD measurements on the Se-poor CIGS films deposited on glass substrates containing different sodium content and different rear contacts.

Sample (Mo on various glass)	CIGS(112)		CIGS (220/204)			*x*
	*2θ* [°]	*I* [counts]	*2θ* [°]	*I* [counts]	I(220/204)/I(112)	
Boro/Mo	26.91	3635	44.56	885	0.24	0.32
Boro/MoNa	26.93	3212	44.54	810	0.25	0.34
Dia/Mo	26.75	6115	44.35	1542	0.25	0.12
SLG/Mo	26.75	7127	44.38	1834	0.26	0.13
SLG/TiN/Mo	26.78	7096	44.41	1883	0.26	0.16
SLG/TiN/MoNa	26.75	6868	44.36	1556	0.23	0.13
SLG/SiN/Mo	26.76	6947	44.39	1939	0.28	0.14
SLG/SiN/MoNa	26.76	7441	44.38	1818	0.23	0.13

The intensity ratio of the CIGS (220/204) peak to the CIGS (112) peak was also calculated for comparison. *x* is the GGI value derived from Eq. 6.3.

As reported in our previous publication^18^, the absorbers formed by the two-step sputtering-selenisation process are consisted of a bilayer structure: i.e., a Ga-rich CGS layer near the back contact and an In-rich CIS layer near the CdS buffer^18^. The final Cu(In_1−x_Ga_x_)Se_2_ film was formed through the interdiffusion between the CGS and CIS layers. However, the diffusion rate of Ga is much lower than that of In in the absorber^17,21^. Hence, CIS and CGS phases are observed from XRD measurement. As the lattice constant increases linearly with reducing Ga content (*x*), the XRD diffraction peaks of the CIS phase will shift to lower angles compared to the CGS phase. The dashed lines in Figs 5 and 6 at 2θ = 26.65° and 27.65° are the (112) reflection position of the single CIS and CGS layers, respectively^21^. The *x* value in Cu(In_1−x_Ga_x_)Se_2_ is derived from the *2θ* value of the CIS (112) reflection in the XRD pattern, using the following equation^29^:

3 $${\rm{x}}=\frac{(y-26.65)}{0.24}\times 0.3=(y-26.65)\times 1.25$$

where *y* is the *2θ* value of the CIGS (112) peak. The calculated *x* values are listed in Table 2. It is evident from Table 2 that the CIGS absorbers formed on borosilicate glass substrates show the highest *x* values, which are closer to the GGI ratio in the films measured by XRF. In contrast, CIGS films formed directly on the SLG substrate without any diffusion barrier layer show the lowest *x* value. The diffusion of Na from the SLG substrate into the CIGS absorber hinders the In and Ga inter-diffusion, and thus reduces the Ga content within the front region of the CIGS absorber. Both TiN and SiN barrier layers can hinder the Na diffusion, and thus promote Ga diffusion to the front. The lower *x* values of the samples deposited on Na-containing glass substrates indicate lower Ga contents near the front surface of the CIGS films^18,19^.

### Se-rich CIGS absorber

Another set of Se-rich (≥57 at.%) CIGS absorbers was prepared using a CuGa and In multilayer precursor with a thickness of about 525 nm. The CGI and GGI ratios in all samples were maintained at around 0.93 and 0.30, respectively. Properties of this Se rich absorber formed on various type of back electrodes are discussed below.

#### Morphology

**Figure 7:**
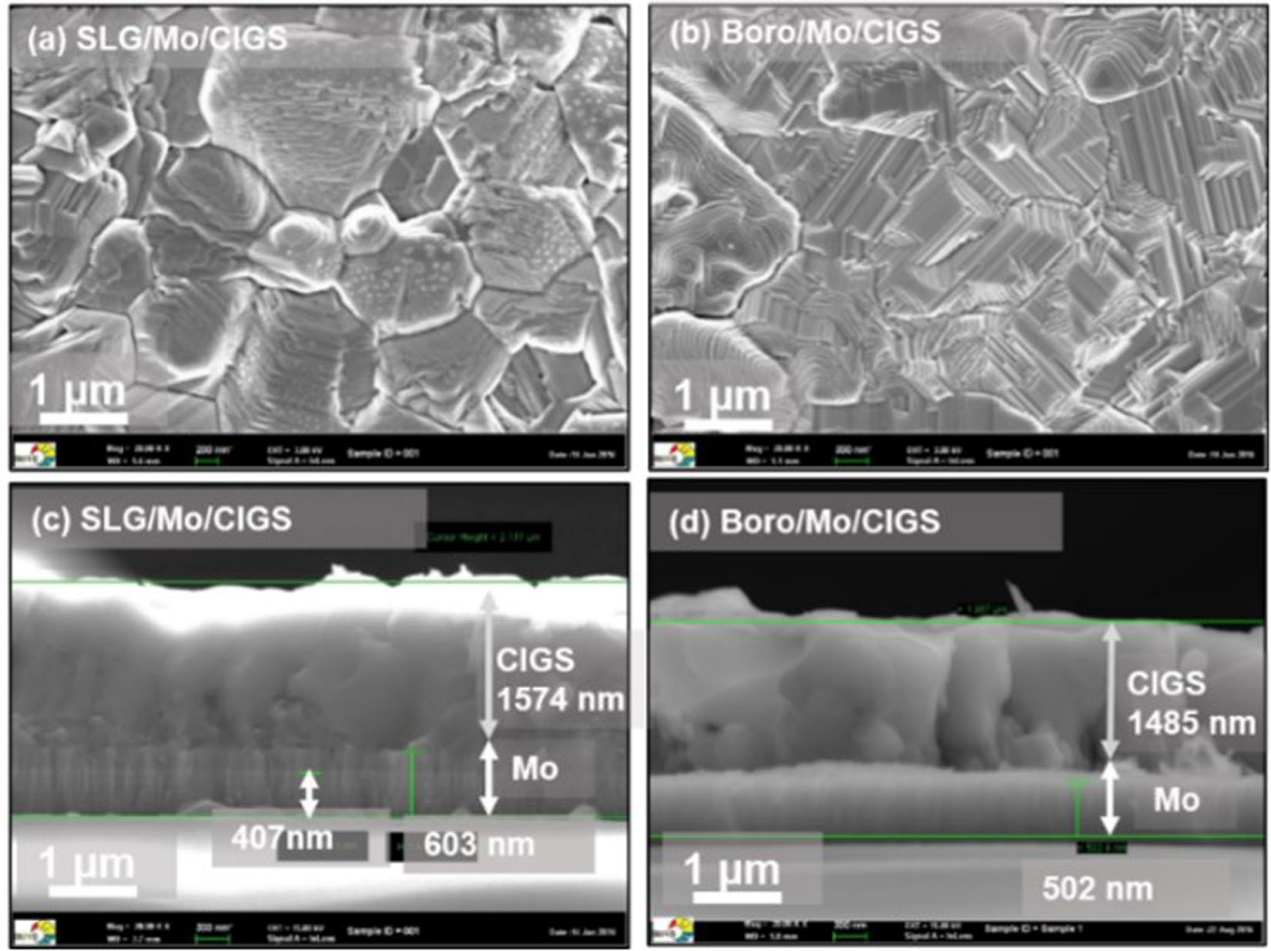
SEM surface and cross-sectional images of Se-rich CIGS absorbers formed on SLG/Mo (**a,c**) and Boro/Mo (**b,d**) substrates.

SEM images of the Se-rich CIGS absorber formed on SLG/Mo and Boro/Mo substrates are shown in Fig. 7. In the SEM surface images (Fig. 7(a)) white sodium selenide precipitates are seen on the SLG/Mo samples (which have a high Na content in the CIGS absorber due to Na diffusion from the substrate into the absorber). In contrast, the samples deposited on Na-free Boro/Mo substrate showed compact and well faceted grains with clear grain boundaries (Fig. 7(b)). SEM measurements also reveals a thick MoSe_2_ layer formation between the Mo and the CIGS layer in the sample deposited on SLG/Mo substrate. The measured MoSe_2_ thickness (~200 nm) is comparable to the estimated value using Eq. 3 (see Table 3).

**Table 3:** Summary of XRF measurements of Se-rich CIGS absorbers prepared usingdifferent rear contacts and glass substrates.

Sample number	CGI	GGI	SeC	SeGI	Se/M	Mo	MoSe_2_	CIGS
						[nm]	[nm]	[nm]
Boro/Mo	0.94	0.31	2.44	2.29	1.18	573	81	1629
Boro/MoNa	0.91	0.30	2.48	2.26	1.18	745	75	1599
SLG/Mo (500 nm)	0.93	0.31	2.72	2.53	1.31	548	140	1680
SLG/Mo (900 nm)	0.91	0.30	2.74	2.49	1.30	848	133	1703
SLG/TiN	0.91	0.30	2.67	2.44	1.28	513	122	1673
SLG/TiN/MoNa	0.93	0.31	2.73	2.53	1.31	775	140	1681
SLG/SiN/Mo	0.93	0.29	2.32	2.16	1.12	511	46	1492
SLG/SiN/MoNa	0.93	0.31	2.37	2.20	1.14	765	57	1514

The same method as in Table 1 was used to calculate the MoSe_2_ thickness and the CIGS thickness.

#### Chemical composition

Table 3 summarises the XRF measurement results of Se-rich CIGS absorbers formed on various rear contact structures. It should be noted that the measured Se/M ratio, MoSe_2_ layer thickness, and the thickness of the Se-rich CIGS absorber are quite different from the values of samples with Se-poor CIGS absorber. The results can be classified into two groups, based on the Na concentration: (i) absorbers with low or negligibe Na concentration, for example SLG/SiN/Mo and Boro/Mo (and also with optional Mo:Na capping layer) rear contacts, and (ii) absorbers with high Na concentration, for example SLG/Mo (500 and 900 nm) and SLG/TiN/Mo(Mo:Na) samples. From the XRF measurements, the values of Se/M ratio, MoSe_2_ thickness and absorber thickness of the low-Na samples were observed to be lower than those of high-Na samples. However, for the Se-poor samples, as discussed in section 3.1, no significant difference is found in the thickness of both the CIGS layer and the intermediate MoSe_2_ layer, regardless of the rear contact structure. Thus, a strong correlation between the Se and Na contents and their influences on the growth of the CIGS absorber and the MoSe_2_ thickness is observed. This observation suggests that presence of excess Na in the CIGS absorber can enhance the Se/M ratio in the CIGS absorber, which further promotes the MoSe_2_ growth, as well as increases the thickness of the CIGS absorber.

#### Microstructure

**Figure 8:**
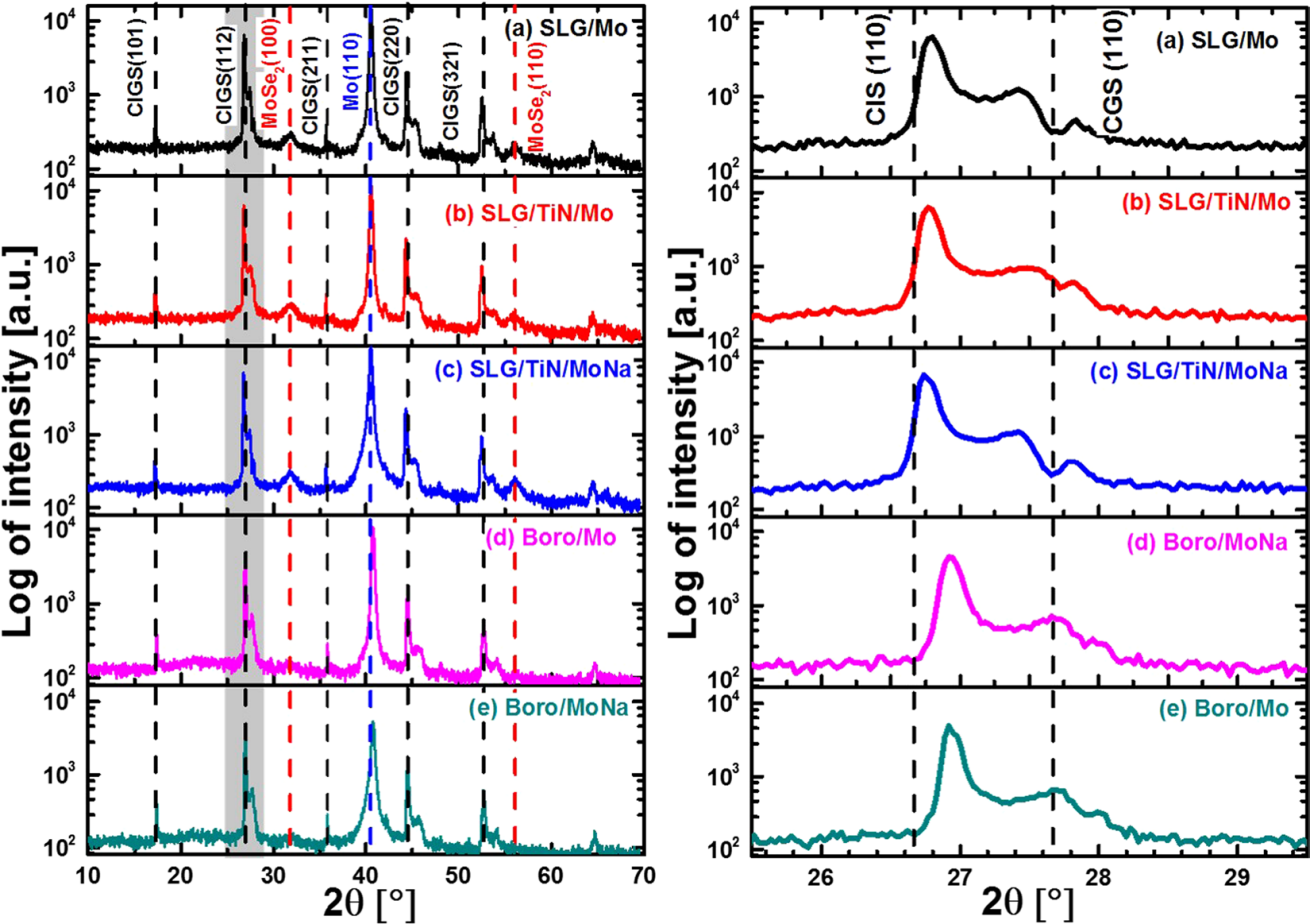
Full range XRD patterns (10–70°) and CIGS (112) peak profiles of Se-rich CIGS absorbers deposited on different rear contacts: (**a**) SLG/Mo, (**b**) SLG/TiN/Mo, (**c**) SLG/TiN/MoNa, (**d**) Boro/Mo and (**e**) Boro/MoNa. The two MoSe_2_ peaks are highlighted by red dashed lines.

The XRD patterns of Se rich CIGS absorbers deposited on different rear contacts are shown in Fig. 8. All of these CIGS films show a polycrystalline structure. The intensity ratio I(220/204)/I(112) of the samples on SLG substrates is higher than that of the samples on borosilicate glass substrates (see Fig. 8). Hence it can be concluded that, in the case of Se-rich CIGS films, Na diffusion from the glass substrate enhances the growth of CIGS grains in the (220/204) orientations. As shown in Table 4, the estimated Ga content (*x* value in Eq. 3) of the samples on borosilicate glass is higher than that of the samples on SLG substrates, as the CIS and CGS interdiffusion is delayed by the presence of Na. However, the Ga content near the CIGS front surface is reduced by a TiN barrier layer for the samples deposited on SLG substrates, which can be ascribed to the suppressed Na diffusion by the TiN barrier from the glass substrate to the CIGS absorber.

**Table 4:** XRD measurement results of Se-rich CIGS absorbers formed on different glass substrates and different back contacts.

Sample	CIGS			x	MoSe_2_
	(112)	(220/204)	I(220/204)/I(112)		I(100)/I(110)
	2θ [°]	2θ[°]			
SLG/Mo	26.80	44.39	0.32	0.20	1.41
SLG/TiN/Mo	26.78	44.39	0.32	0.16	2.28
SLG/TiN/MoNa	26.75	44.38	0.29	0.13	2.03
Boro/Mo	26.93	44.54	0.25	0.35	—
Boro/MoNa	26.93	44.57	0.25	0.35	—

The intensity ratio of CIGS (220/204) peak to CIGS (112) peak of CIGS absorber was also derived for comparison. *x* is the GGI value derived from Eq. 2. The intensity ratio of (100) to (110) peak of the MoSe_2_ layer are also calculated.

From Fig. 8(e), it can be seen that for the samples formed on borosilicate glass substrates with an additional Mo:Na layer as extrinsic Na source, no MoSe_2_ diffraction signal was detected from the XRD measurement, which is due to the low amount of MoSe_2_ at the Mo/CIGS interface. The low growth rate of the MoSe_2_ layer can be explained by insufficient Na_2_Se intermediate compound formation at the rear surface. As discussed in our previous publication^18^, for the CIGS films with higher Na diffusion from the substrate, it is observed to have higher Na and Se content in the form of Na_2_Se_x_ compound at the Mo/CIGS surface^14,18,30^. Additionally, from XRF measurements (Table 4), the observed MoSe_2_ thickness was significantly higher for Na-rich glass substrates than for Na-deficient ones. Thus, it is reasonable to conclude that the growth of the MoSe_2_ layer can be accelerated by Na_2_Se_x_. In other words, Na_2_Se_x_ can be considered as a catalyst to form a thick MoSe_2_ layer.

If all the Na from the 200 nm thick Mo:Na capping layer is assumed to be uniformly distributed in the completed CIGS solar cell, the Na content in the CIGS absorber would be about 0.5 at.%, which is higher than the observed Na content in CIGS solar cells formed on SLG/Mo substrates^18^. Thus, it can be concluded that most of the Na atoms diffusing from the Mo:Na layer are trapped within the Mo grains and cannot easily diffuse into the CIGS film.

From the XRD measurements, the value of the MoSe_2_ peak intensity ratio I(100)/I(110) was calculated, see Table 4. Since this value is higher than 1.0, this indicates a preferred grain orientation of the MoSe_2_ layer in the <100> direction. It has been reported that a MoSe_2_ grain orientation in the <110> direction reduces the contact resistance between the Mo and the CIGS layers, and enhances the adhesion of the CIGS layer to the rear contact^13,31–33^. In addition, the conductivity of (100)-orientated MoSe_2_ grains, aligned in parallel to the Mo rear contact surface, is two times lower than that of (110)-orientated MoSe_2_ films, which have grains that are perpendicular to the Mo rear contact. Therefore, the MoSe_2_ intermediate layer formation needs to be adjusted by optimizing the selenisation process and by controlling the Na diffusion to obtain the preferred (110) orientation.

## Discussion and Conclusion

### Dependence of morphology and microstructure of the CIGS absorber on the Mo-coated glass substrates

The sodium diffusion either from the glass substrate or from the Mo rear contact plays a large role in the grain growth of the CIGS absorber layer, as the sodium can promote the grain growth of the CIGS absorber layer. Na diffusion to the front surface of the CIGS absorber is beneficial for forming large crystallites near the front surface. The CuInSe_2_ on the CIGS surface can be formed through the reaction of either CuSe_2_ or CuSe with InSe. However, the grain size of Cu_2_Se is larger than that of CuSe^34,35^. The sodium selenide formed at the front surface of the CIGS can act as a Se source to promote the formation of larger CuSe_2_ grains, resulting in large CIS grains near the surface. At the same time, the diffused Na suppresses the CuInSe_2_ formation via reaction of smaller grain CuSe with InSe. The CIGS absorber formed on the TiN modified Mo rear contact shows the best uniformity in grain size, indicating the good control of Na diffusion from the SLG glass substrate by utilizing the TiN diffusion barrier layer.

All the CIGS films show a bilayer microstructure verified by XRD measurement, i.e., a Ga-rich CGS layer near the back contact and an In-rich CIS layer near the CdS buffer. The growth of the CuInSe_2_ and CuGaSe_2_ grains across the film thickness requires different formation energy, time and temperature during the selenisation process. The final Cu(In_1−x_Ga_x_)Se_2_ chalcogenide film is formed via inter-diffusion between the CGS and CIS layers. However, the Na diffusion from the substrate affects the inter-diffusion between the CGSand CIG layers. The CIGS absorbers deposited on borosilicate glass substrates show the highest *x* values, which are closer to the GGI ratio in the films measured by XRF. In contrast, CIGS films formed directly on the SLG substrate without any barrier layer show the lowest *x* value. The Na diffusion from the SLG substrate into the absorber reduces the In and Ga inter-diffusion, and thus reduces the Ga content at the front surface of the CIGS absorber. The TiN and SiN barrier layers can hinder the Na diffusion^18^, and thus promoted Ga diffusion to the front surface. The lower *x* values of the samples deposited on Na-containing glass substrates indicate a lower Ga content at the front surface of the CIGS absorber.

### Formation of MoSe_2_ intermediate layer

The precise control of the MoSe_2_ thickness and its crystal orientation are the most important factors determining the solar cell efficiency. Based on this study, the formation of the MoSe_2_ layer depends on both Na diffusion from the substrate and the Se content, which are related to the sodium content on the substrate, back contact structure and the selenisation process.

All selenisation processes in this study were conducted at a substrate temperature of 580 °C for a duration of 12 minutes. From the experiments, it was observed that there is no difference in the thickness of the MoSe_2_ layer in the Se-poor CIGS absorbers, while the thickness of the MoSe_2_ layer varies significantly in the Se-rich CIGS absorbers. In particular, for the Se-poor absorber, the Se is fully consumed by the metallic CIG precursor to form the CIGS absorber. Thus, the formation of MoSe_2_ is inhibited due to the lack of Se in the absorber forming environment. In this situation, the CIGS/Mo interface is stable even during long-time annealing in a Se-deficient condition, regardless of the amount of Na diffusing from the substrate.

In contrast, in Se-rich absorbers, Na_2_Se forming at both the Mo/CIGS interface and the CIGS grain boundaries tend to increase the MoSe_2_ formation probability. In this case, the excessive Se reacts with the Na diffusing from the substrate to form Na_2_Se_x_, which is usually located at the CIGS/Mo interface as a Se source and catalyses the MoSe_2_ formation. When there is insufficient or no Na available to form Na_2_Se_x_, it is less probable to form MoSe_2_ by the direct reaction between Se and Mo atoms. Hence, a very thin MoSe_2_ layer is found in the Se-rich samples made on rear contacts with low Na concentrations, for example the Na-free substrates and SLG substrates with SiN acting as a good Na diffusion barrier layer. A thick MoSe_2_ layer is only observed in samples with high Na diffusion from the substrate, and its thickness increases with increasing Na content in the glass substrate. However, if the MoSe_2_ layer gets too thick, the series resistance tends to increase and the solar cell efficiency was found to decrease.

In this study, the influence of the sodium diffusion from the substrate on the properties of two-step process formed CIGS absorber using elemental Se vapour are investigated. Using a TiN layer on SLG glass as a barrier layer to adjust Na diffusion, the CIGS film shows the best uniformity in grain size, indicating the good control of Na diffusion from the SLG glass substrate. The TiN barrier layers can partially reduce the Na diffusion, and thus enhance Ga and In inter-diffusion to form large CIGS grains. We also find that both Na and Se play crucial roles in the formation of the MoSe_2_ intermediate layer. A Se-rich atmosphere is required to grow a thick MoSe_2_ layer, while a Se-poor CIGS formation process leads to a thin MoSe_2_ layer, regardless of the Na content in the substrates. Since the series resistance of the CIGS solar cells increases with increasing MoSe_2_ layer thickness, an intermediate MoSe_2_ layer thickness will give the best device performance. Thus, the Se flow in the two-step CIGS formation process must be sufficiently high to obtain high-efficiency CIGS solar cells.
